# Study on the Efficacy of Peracetic Acid Disinfectant (Type III) on Gastrointestinal Endoscopy Disinfection

**DOI:** 10.1097/SLE.0000000000000921

**Published:** 2021-03-12

**Authors:** Nan Zhang, Jianqiang Guo, Lan Liu, Honglei Wu, Jiaoyang Gu

**Affiliations:** Department of Gastroenterology, The Second Hospital of Shandong University, Jinan city, Shandong Province, Peoples Republic of China

**Keywords:** gastrointestinal endoscopy, disinfectants, peracetic acid disinfectant (Type III)

## Abstract

**Purpose:**

The purpose of this study was to evaluate the disinfection efficacy of peracetic acid disinfectant (Type III) on gastrointestinal endoscopy.

**Methods:**

Endoscopes were disinfected, respectively, by 2% glutaraldehyde and peracetic acid disinfectant (Type III) according to the procedures stipulated by the 2016 version of “Regulation for cleaning and disinfection technique of flexible endoscope,” then samples were collected through biopsy channel at the specified steps. The bacterial count and pathogenic bacteria of these samples were detected, and hepatitis B virus surface antigen, hepatitis C virus antibody, and Treponemiapallidum antibody were detected by chemiluminescent microparticle immunoassay in peracetic acid disinfectant (Type III) group. The samples from the peracetic acid disinfectant (Type III) group were collected for 5 days continuously.

**Results:**

In total, 56 gastroscopes and 16 colonoscopes were disinfected by 2% glutaraldehyde (GA Group), 46 gastroscopes, and 15 colonoscopes were disinfected by peracetic acid disinfectant (Type Ⅲ) (PAA Group). After disinfection, the bacterial count was significantly reduced in the 2 groups (*P*<0.05). In terms of the qualified rate of gastroscopes and total qualified rate, the PAA Group was better than GA Group [the qualified rate of gastroscopes: 97.83% (45/46) vs. 92.86% (52/56), *P*>0.05; total qualified rate: 98.36% (60/61) vs. 94.44% (68/72), *P*>0.05], the qualified rate of colonoscopes in the 2 groups were both 100.00% (15/15, 16/16). After disinfecting by peracetic acid disinfectant (Type Ⅲ), hepatitis B virus surface antigen, anti-hepatitis C virus, and Treponemiapallidum antibody were negative. In term of colonies number detected for 5 days continuously, there was no significant difference at different collection steps (*P*>0.05).

**Conclusions:**

Peracetic acid disinfectant (Type III) can be well applied to clinical with meeting the standard of high-level disinfection for gastrointestinal endoscopy, and after disinfecting by peracetic acid disinfectant (Type III), there was no obvious bacterial residue in the biopsy channel.

With the development of medicine, the essential role of gastrointestinal endoscopy in the diagnosis and treatment of digestive diseases has been widely recognized. It is reported that >10 million cases of endoscopic examinations and treatments each year have been carried out in the United States.^1^ However, gastrointestinal endoscopes are invasive instruments with a sophisticated structure, which are at increased risks of developing nosocomial infections and virus transmission because of nonstandard operations during cleaning and disinfection procedures and improper use of disinfectants.^2^ A number of studies have shown that during the diagnostic and therapeutic interventions, the incidence rate of bacterial cross-infection and transmission of virus-like hepatitis B virus (HBV) or hepatitis C virus (HCV) increase because of nonstandard reprocessing procedures.^3^–^7^ The most widely used disinfectant is 2% glutaraldehyde in China nowadays,^8^ but it is generally found that it may form hidden dangers to the health care of medical staves and patients.^9^ In order to find more efficient and safe disinfectants, this trial has been carried out.

## METHODS

### Study Subjects and Materials

This study was designed as a prospective study. This study was approved by the Institutional Review Board of Second Hospital of Shandong University. Endoscopes (Olympus Corporation, Tokyo, Japan) examined by out-patients and in-patients of the Second Hospital of Shandong University from March 2017 to December 2017 were included in the present study. Automated endoscope reprocessors (Olympus Corporation, Tokyo, Japan) containing 2% glutaraldehyde and peracetic acid disinfectant (Type III), respectively, were used. Peracetic acid disinfectant (Type III) is a dual package, containing A and B agents, both of which are liquid formulations. A and B were mixed uniformly for 5 minutes before using, keeping the concentration at 1500 mg/L. MicroScan WalkAway-96 Plus system from Siemens (Germany) was used for bacterial identification and susceptibility testing. ARCHITECT i2000_SR_ system of Abbott (USA) was used for detecting virus through chemiluminescent microparticle immunoassay (CMIA).

### Methods

**FIGURE 1:**
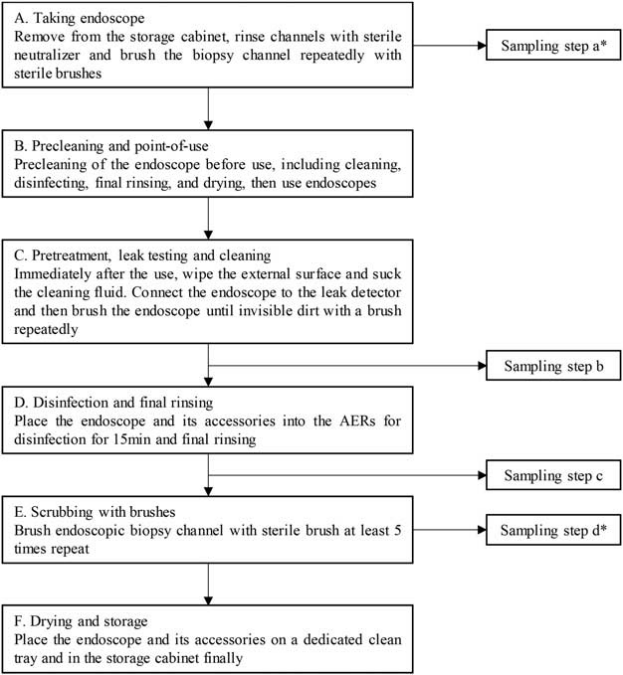
Steps of endoscopic cleaning, disinfecting, and sampling. *Selected endoscopes disinfected with peracetic acid disinfectant (Type III), and each endoscope was continuously tested for 5 days. The remaining endoscopes did not perform this procedure. AER indicates automated endoscope reprocessors.

The protocol of cleaning, disinfection, and sampling: Endoscopes were cleaned and disinfected on the basis of the procedures stipulated by the 2016 version of “Regulation for cleaning and disinfection technique of flexible endoscope.” The disinfection was kept within the effective concentration range (>1000 mg/L) and would not be used >7 days at 1 time. Specific cleaning, disinfection, and sampling procedures are shown in Figure 1. In steps b and c, 50 mL sterile neutralizer was injected into the biopsy channel and the whole amount of eluent was collected and thoroughly mixed for testing. Sterile scissors were used in steps c and d to cut the brush down into sterile sample cups containing neutralizing agent, then the samples were mixed well and submitted for testing.

#### Bacterial Culture and Colony Count

According to the methods ruled by GB 15982-2012 “Hygienic standard for disinfection in hospitals,” 1 mL of the above eluting liquid was inoculated onto a flat plate. Then 15 to 20 mL of the melting nutrient agar medium was poured at 40 to 45°C per dish. The remaining eluent was aseptically filtered and concentrated on a membrane filter (0.45 μm). The filter was inoculated onto a solidified nutrient agar plate without any air bubbles. The samples were all cultured for 48 hours at 36±1°C in the incubation chamber. Finally, count the colonies. Preliminarily determine whether it was pathogenic bacteria according to the characteristics and types of colonies. The suspected bacterial colonies were inoculated into the sample plates according to the operating instructions of MicroScan WalkAway-96 Plus system to identify the bacterial species and the test results were recorded.

#### Quantitative detection of hepatitis B virus surface antigen (HBsAg), HCV antibody, and Treponemiapallidum antibody (TP-Ab)

Three milliliter of the above eluent was sampled to ARCHITECT i2000SR system and detected by CMIA according to the operation procedures. Samples were collected and tested only in the endoscopic procedures performed by patients who were positive for HBV, HCV, or TP-Ab.

### Evaluation Standard

#### Bacteria and Pathogens

According to the 2016 version of “Regulation for cleaning and disinfection technique of flexible endoscope,” the qualified endoscope disinfection standard is no >20 cfu/ piece of the colony, and no pathogenic bacteria can be detected.

#### HBsAg, HCV Antibody, and TP-Ab

It should be negative.

### Statistical Analysis

SSPS 22.0 statistical software for statistical analysis, counting data using χ^2^ test or Fisher exact test, the measurement data complied with the normal distribution and was showed as *M* (P_25_, P_75_), using Mann-Whitney *U* test to determine whether there was a difference in the number of colonies between the 2 groups, Wilcoxon signed-rank test was used to determine whether there was a difference in the number of colonies before and after disinfection, Friedman test was used to analyze the residual bacteria adhesion on the surface of endoscopic biopsy pipeline after disinfected by peracetic acid disinfectant (Type III). Whether the *P*-value was statistically significant difference was determined by 95% confidence interval (CI) range.

## RESULTS

### Disinfection Qualified Rate (Colony Detection)

In this part, a total of 102 gastroscopies and 31 colonoscopies were selected. In total, 56 gastroscopies and 16 colonoscopies were disinfected by 2% glutaraldehyde (GA group), 46 gastroscopies, and 15 colonoscopies were disinfected by peracetic acid disinfectant (Type III) (PAA Group). After disinfection, the bacterial count of gastroscopes and colonoscopes was significantly reduced in both 2 groups (Table 1). No pathogenic bacteria was detected.

**TABLE 1:** Comparison of Colony Counts Before and After Disinfection (cfu/Piece)

	Gastroscopies (n=102)				Colonoscopies (n=31)			
	GA (n=56)	PAA (n=46)	*z*	*P*	GA (n=16)	PAA (n=15)	*z*	*P*
Predisinfection	0 (0.00, 50.00)	0 (0.00, 28.75)	−1.343	0.179	0 (0.00, 81.25)	5 (0.00, 65.00)	−0.520	0.654
Postdisinfection	0 (0.00, 0.00)	0 (0.00, 0.00)	−1.183	0.237	0 (0.00, 0.00)	0 (0.00, 0.00)	−0.968	0.770
*z*	−4.112	−3.019			−2.207	−2.524		
*P*	0.000	0.003			0.027	0.012		

GA indicates 2% glutaraldehyde disinfectant; PAA, peracetic acid disinfectant (Type III).

The qualified rate of gastroscopes in GA Group was 92.86% (52/56), which of colonoscopes was 100% (16/16), and the total qualified rate was 94.44% (68/72). With regard to PAA Group, the qualified rates were 97.83% (45/46), 100% (15/15), and 98.36% (60/61), respectively (Table 2). There was no statistically significant difference in the disinfection qualification rate of gastroscopes, colonoscopes, or total qualification rate of the 2 disinfectants (*P*>0.05).

**TABLE 2:** Comparison of Qualified Rates of 2 Disinfectants [n (%)]

		Gastroscopies		Colonoscopies	
Groups	n	Qualified	Unqualified	Qualified	Unqualified
GA	72	52	4	16	0
PAA	61	45	1	15	0

GA indicates 2% glutaraldehyde disinfectant; PAA, peracetic acid disinfectant (Type III).

The total qualified rate of PAA Group (*P*_t_) was 98.36% and the total qualified rate of GA Group (*P*_c_) was 94.44%. According to the calculation formula of noninferiority test *t*=(*d*+δ)/*S*_d_ $\left[d=P_{t}-P_{c},\;\delta =10\%,\;\;S_{d}=\sqrt{\frac{X_{1}+X_{2}}{n_{1}+n_{2}}\left(1-\frac{X_{1}+X_{2}}{n_{1}+n_{2}}\right)\left(\frac{1}{n_{1}}+\frac{1}{n_{2}}\right)}\right]$, we could get that *t*=4.205, *P*<0.05. It can be inferred that peracetic acid disinfectant (Type III) was not inferior to 2% glutaraldehyde disinfectant. According to 1-sided (1−α), the CI (*C*_L_, ∞) of T−C was (−0.015, ∞) (*C*_L_ = d−1.64 *S*_d_), which is completely within (−δ, ∞), the noninferiority conclusion was established.

### HBsAg, HCV Antibody, and TP-Ab Results

A total of 24 gastroscopies and 21 colonoscopies were selected for being disinfected by peracetic acid (Type III) in this part, including 19 gastroscopies and 17 colonoscopies of HBsAg-positive, 4 gastroscopies and 3 colonoscopies of HCV antibody-positive, and 1 gastroscopy and 1 colonoscopy of TP-Ab positive. Samples were collected at steps b and c and detected through CMIA. The results showed that HBsAg, HCV antibody, and TP-Ab of gastroscopies and colonoscopies were all negative after disinfection.

### Residual Bacteria on Surface of Endoscopic Biopsy Channels After Disinfection of Peracetic Acid (Type III)

In this part, 12 gastroscopies used in daily work were selected and all were disinfected by peracetic acid (Type III). The results of each endoscope detected for 5 consecutive days indicated that there was little or no residual bacteria attachment on the inner wall of the endoscopic biopsy channel after the disinfection with peracetic acid, and there was no correlation with sampling procedures or using the time (Table 3).

**TABLE 3:** Residual Bacteria on the Surface of Endoscopic Biopsy Channels After Disinfection (cfu/Piece)

	D1	D2	D3	D4	D5	χ^2^	*P*
Step a	0 (0, 0)	0 (0, 0)	0 (0, 0)	0 (0, 0)	0 (0, 0)	3.500	0.478
Step d	0 (0, 0)	0 (0, 0)	0 (0, 0)	0 (0, 0)	0 (0, 0)	2.222	0.695

## DISCUSSION

Gastrointestinal endoscopic diagnosis and treatment technology has been developed rapidly. The number of these procedures performed for diagnostic purposes or therapeutic inventions is increasing significantly. It is particularly important to perform endoscopic disinfection and avoid cross-infection in hospitals because of the association between unqualified disinfection of endoscopes and nosocomial infection. A total of 2% glutaraldehyde disinfectant is the most widely used disinfectant at present,^8^ however, with the long-term clinical application and research, many non-negligible negative effects have been recognized. The 2016 version of “Regulation for cleaning and disinfection technique of flexible endoscope” and several research reports^9^ indicated that the glutaraldehyde disinfectant was irritant and sensitive, especially on skin, eyes, otolaryngology and respiratory mucosa, causing dermatitis, conjunctivitis, occupational asthma, even systemic toxicity. In addition, residual glutaraldehyde after disinfection can lead to chemical colitis, abdominal cramps, and even hemorrhagic diarrhea. Glutaraldehyde disinfectant is easy to form induration on the endoscope and disinfection equipment, which is difficult to be removed, affecting the lifetime of endoscopic instruments.^9^ Therefore, in recent years, more and more experts have suggested to find safer disinfectants and replace it. Peracetic acid is a strong oxidant with the ability to kill microorganisms such as bacteria, fungi, and viruses by reacting with enzymes, amino acids, nucleic acids, and cleavaging DNA bases and double chains, widely used in surgical instrument sterilization, pharmaceutical, and food manufacturing.^10^

The results showed that both 2% glutaraldehyde and peracetic acid disinfectant (Type III) significantly decreased the colony counts of gastroscopies and colonoscopies after disinfection. The qualified rate of the gastroscopies (97.83%) and the total qualified rate (98.36%) in the peracetic acid group were both higher than that of the glutaraldehyde group (92.86% and 94.44%, respectively). The qualified rate of the colonoscopies in 2 groups was both 100%. It suggests that the 2% glutaraldehyde disinfectant used in this endoscopy center and the peracetic acid (Type III) disinfectant used in this research institute can both achieve the expected disinfection effect on gastroscopies and colonoscopies, but the peracetic acid disinfectant (Type III) performed better than 2% glutaraldehyde disinfectant on the qualified rates, health care for staves, and other aspects.^10^

The research about killing effect of novel peracetic acid disinfectant on common viral such as HBV is still rarely reported in domestic and abroad. In this study, gastroscopies and colonoscopies used by HBsAg, HCV antibody, or TP-Ab-positive patients were randomly selected and normatively disinfected by peracetic acid disinfectant (Type III). Results showed that HBsAg, HCV antibody, and TP-Ab were all negative after disinfection. Thus, the safety of the endoscopes under the standard cleaning and disinfection procedures in this endoscopy center was confirmed.

In this study, 12 gastroscopies used in daily work were randomly selected, each of which was sampled before use and after disinfection with peracetic acid (Type III), and the number of bacterial colonies on the brush was detected. Each gastroscopy was continuously tested for 5 days. The results showed that there were little or no residual bacteria on the brushes, and there was no significantly different in the number of colonies collected at each step or at each time point. Furthermore, there was no correlation between the number of colonies and the progression of endoscopic using time. It can be considered that there was no obvious residual bacteria that could be difficultly removed on the surface of the inner wall of the endoscopic biopsy channel after disinfection with peracetic acid disinfectant (Type III). Some Japanese scholars established in vitro biofilm models for *Staphylococcus aureus* and *Pseudomonas aeruginosa*, and sterilized with different disinfectants. They found that peracetic acid had a faster disinfection effect than other disinfectants such as glutaraldehyde and orthophthalaldehyde, suggesting the good disinfection efficacy of peracetic acid.^11^

However, there are still other deficiencies in the trial, including the small sample size, not related to the killing effect on *Helicobacter pylori*, and the endoscopic corrosion effect has not been conducted yet. Besides, disinfection efficiency is an important aspect. In this trial, we did not compare the time required for the 2 disinfectants to achieve the same disinfection effect. Further exploration is needed.

In conclusion, this study further confirmed that peracetic acid disinfectant (Type III) can achieve effective high-level disinfection from the aspects of bacteria, viruses, and the presence of residual bacteria after disinfection. This study confirmed that the peracetic acid disinfectant (Type III) can efficiently and safely perform endoscopic disinfection, meeting the requirements of the new regulations, with low concentration and less irritation to decontamination personnel, and can be better applied in clinical practice.
